# Bosniak classification of cystic renal masses, version 2019: Is it helpful to incorporate the diffusion weighted imaging characteristic of lesions into the guideline?

**DOI:** 10.3389/fonc.2022.1004690

**Published:** 2022-10-18

**Authors:** Anqin Li, Shichao Li, Yao Hu, Yaqi Shen, Xuemei Hu, Daoyu Hu, Ihab R. Kamel, Zhen Li

**Affiliations:** ^1^ Department of Radiology, Tongji Hospital, Tongji Medical College, Huazhong University of Science and Technology, Wuhan, China; ^2^ Russell H. Morgan Department of Radiology and Radiological Science, Johns Hopkins Medical Institutions, Baltimore, MD, United States

**Keywords:** cystic renal mass, Bosniak classification, magnetic resonance imaging, diffusion weighted imaging, diagnostic performance

## Abstract

**Objective:**

To improve understanding of diffusion weighted imaging (DWI) characteristic of MRI and clinical variables, further optimize the Bosniak classification for diagnosis of cystic renal masses (CRMs).

**Methods:**

This study retrospectively analyzed 130 CRMs in 125 patients with CT or MRI, including 87 patients with DWI (b = 600, 1000 s/mm2). Clinical variables and histopathological results were recorded. Two radiologists in consensus analyzed images of each lesion for the size, thickness of wall, number of septum, enhancement of wall/septum, wall nodule, signal intensity on DWI, calcification, and cyst content. Clinical variables, CT and MRI image characteristics were compared with pathology or follow-up results to evaluate the diagnostic performance for CRMs.

**Results:**

Of the 130 lesions in 125 patients, histological analysis reported that 36 were malignant, 38 were benign, and no change was found in 56 followed-up lesions (mean follow-up of 24 months). The incidences of cystic wall thickened, more septa, measurable enhancement of wall/septum, nodule(s) on CT/MRI, and high signal intensity on DWI were significantly higher in malignant than in benign CRMs (CT: p = 0.005, p < 0.001, p < 0.001, p < 0.001, p < 0.001; MRI: p < 0.001, p < 0.001, p < 0.001, p < 0.001, p < 0.001, p < 0.001). Combination of MRI including DWI features with CT findings showed the highest area under ROC curve (0.973) in distinguishing benign and malignant CRMs.

**Conclusions:**

Incorporating DWI characteristic of CRMs into Bosniak classification helps to improve diagnostic efficiency.

## Introduction

Cystic renal masses (CRMs) are common and are being detected more and more frequently due to the increasing use of cross-sectional imaging (1–3). CRMs can be classified into simple cysts and complicated cystic lesions. Among them, simple cysts do not require any intervention or follow up (4, 5). However, 21% of cystic masses show a complex pattern, making it difficult to identify benign or malignant and subsequent treatment (1, 3, 6).

The CT-based Bosniak classification (version 2005) has been widely used to evaluate CRMs and guide clinical management (4, 7, 8). Because of the superiority in soft tissue resolution and imaging technology, MRI exhibits additional findings in some cases, such as irregular thickened walls, more septa, and enhancement at subtraction imaging (3, 9). Recently, the Bosniak classification (version 2019) has formally incorporates MRI into the classification to help improve the characterization and diagnosis of CRMs (10).

Diffusion weighted imaging (DWI) is a promising noninvasive imaging modality that has been widely used in oncology (11). The American College of Radiology–endorsed Reporting and Data Systems (RADS) are expert-devised guidelines for the evaluation and interpretation of disease-oriented imaging studies. Several RADS for cancer imaging, such as prostate, vesical, and breast, have included DWI for evaluating tumor biological behavior (12–14). Many studies showed that DWI can provide additional histological features inside the renal tumors, including cystic masses, and help distinguish benign from malignant tumors (3, 5, 15, 16). However, the Bosniak classification (version 2019) did not incorporate DWI into the guideline.

In addition, there are some patient-related clinical factors that may be helpful in identifying benign and malignant, such as body mass index (BMI) of patients, but are not included in the classification criteria (2, 3, 5). Therefore, the Bosniak classification for the assessment of CRMs remain needs to be further investigated.

The purpose of this study was to explore whether incorporating the DWI characteristic of CRMs into the Bosniak classification (version 2019) can help improve the diagnostic accuracy.

## Materials and methods

### Study population

Radiology Information System (RIS) was searched for patients diagnosed with CRMs in our hospital from September 4, 2012, to April 9, 2019. Demographic and clinical data in electronic medical records and imaging reports were recorded, including patient gender, age, BMI, location and the largest diameter of the cystic mass. The inclusion criteria were as follows (1): Patients preliminarily diagnosed as CRMs by ultrasound or CT (2); Patients had performed CT or MRI examination; and (3) Patients received surgical treatment or follow-up. The exclusion criteria were as follows (1): Patient had no CT or MRI examination images before treatment (2); Demographic and clinical data were incomplete; and (3) Patients were lost to follow-up.

### CT examination

The unenhanced CT scan were performed at our institution using one of the following multi-detector CT scanners: Discovery CT750 (GE Healthcare, Milwaukee), Aquilion ONE 320CT (Toshiba Medical Systems, Japan), and Brilliance iCT (Philips Medical Systems, Netherlands). The contrast enhanced CT, 1.5 ml/kg of contrast material (Ultravist 370, Bayer Schering Pharma, Germany) was injected into an antecubital vein at a rate of 3.0 ml/s *via* a pump injector. The cortical phase, parenchymal phase and pelvic phase images were obtained at about 21 secs, 50 secs and 280 secs after the start of injection. The scanning parameters were as follows: 100 to 120  kV, 180 to 210  mA, 1 to 5  mm slice thickness reconstruction.

### MRI examination

MRI examinations were performed using one of the following scanners: Discovery MR750 3.0 T scanner (GE Healthcare, Milwaukee), Brivo MR360 1.5 T scanner (GE Healthcare, GE Healthcare, Milwaukee), and Magnetom Skyra 3.0 T MR scanner (Siemens Healthcare, Germany). The routine renal protocol included the following sequences (1): breath-hold coronal single shot fast spin echo (SSFSE) T2WI: TR/TE, 1840/68 ms; FOV, 340 × 340 mm; matrix, 288 × 288; slice thickness, 5 mm; intersection gap, 1 mm (2); transverse liver acquisition with volume acceleration (LAVA) T1WI: TR/TE, 3.8/1.7 ms; FOV, 400 × 400 mm; matrix, 260 × 210; slice thickness, 4 mm (3); transverse respiratory-triggered T2WI: TR/TE, 8571/69 ms; FOV, 360 × 360 mm; matrix, 320 × 320; slice thickness, 5 mm; intersection gap, 1 mm.

Transverse DWI with respiratory-triggered was obtained by using a single-shot echo-planar imaging (SS-EPI) before the injection of contrast agents. The scanning parameters were as follows: TR/TE 4000/112 ms, FOV 360 × 360 mm, matrix 128 × 160, section thickness 5 mm, intersection gap 1 mm, and bandwidth 250 KHz/pix. Two b values including 600 and 1000 s/mm^2^ were used in three orthogonal diffusion directions. The literature shows that the b value of DWI sequence selection of 600-1000 is suitable for abdominal organs including kidney. And by comparing two different b-value images to highlight the diffusion characteristics of water molecules in the lesions, it is helpful to distinguish benign from malignant (17–19).

Contrast enhanced MRI was performed with transverse LAVA T1WI sequence. 0.1 mmol/kg of contrast material (Gd-DTPA, Bayer Schering Pharma, Germany) was injected into an antecubital vein at a rate of 1.5 ml/s *via* a pump injector. The cortical phase, parenchymal phase and pelvic phase images were obtained at 20-30 secs, 55-60 secs and 180-240 secs after contrast injection, each breathhold acquisition for 16 secs.

### Imaging analysis

The CT and MRI images of patients were reviewed by two radiologists (with 9 and 24 years of diagnostic experience, respectively), who were blinded to previous imaging reports and pathological results. If there were disagreements, the discrepancies were solved in consensus. CRMs were evaluated according to thickness of cyst wall (thin: < 4 mm; thickened: ≥ 4 mm), number of septum (less: < 4; more: ≥ 4), enhancement of wall/septum (perceptible: < 4 mm; measurable: ≥ 4 mm), one or more nodule(s) (≥ 4 mm convex protrusion with obtuse margins, or a convex protrusion of any size that has acute margins), signal intensity (low signal intensity or high signal intensity) of solid component (wall/septum/nodule) on DWI, have calcification of any type, and characteristic of intracapsular density/signal intensity (homogeneous or heterogeneous).

### Reference standards

Based on histopathological diagnosis or clinical follow-up the performance of CT/MRI evaluation, the CRMs were divided into benign or malignant. The diagnostic accuracy for differentiation between benign and malignant CRMs was assessed by patient demographics, mass features, and characteristics of lesion on CT and MRI including DWI.

### Statistical analyses

Statistical analyses were performed with statistical software (PASW version 19.0, SPSS). The Student’s t test was used to compare the age between benign and malignant CRMs. The Chi-squared test was used to compare the gender, BMI of patients, tumor size, location, and the characteristics of lesion on CT and MRI including DWI between benign and malignant CRMs. Receiver operating characteristic (ROC) analysis was constructed to determine the best diagnostic accuracy based on the Youden index. The area under ROC curve (AUC) of CT and MRI including DWI in the evaluation of CRMs was estimated. *P* < 0.05 was considered to have statistical significance.

## Results

### Study cohort

**Figure 1:**
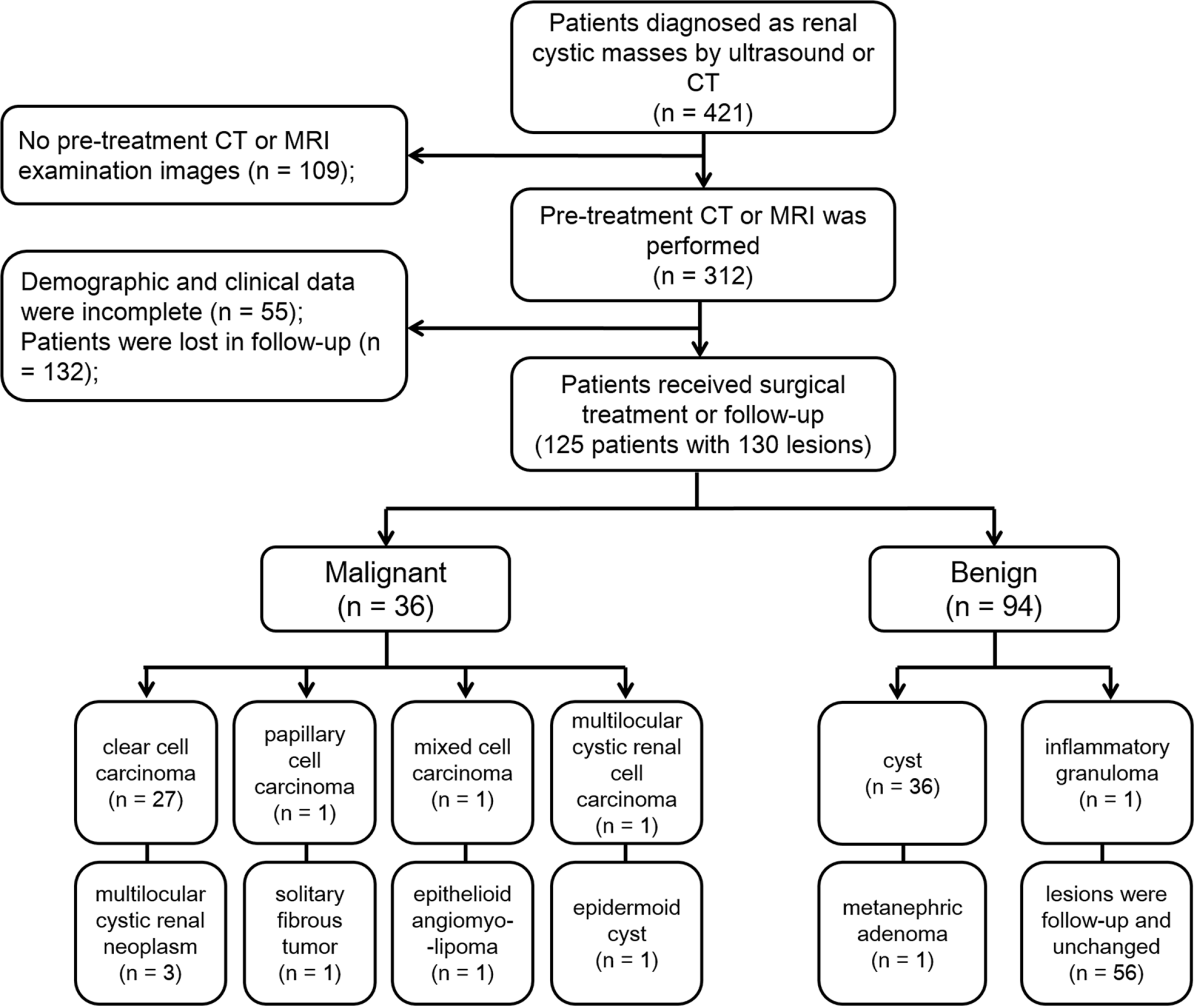
Flowchart of patient inclusion and exclusion.

A study sample flow diagram is provided in **Figure 1**. Finally, our study included 125 patients with 130 lesions. Thirty lesions were confirmed to be malignant by pathology after surgery. Six lesions were confirmed to be borderline tumors by pathology. According to the European Association of Urology guidelines, although these lesions are benign/intermediate/low malignant, they have malignant potential and require surgical resection, so we included them in the malignant group. Thirty-eight lesions were proved to be benign by pathology after surgery. The remaining 56 lesions were classified as benign lesions, which were classified as class I or II according to Bosniak classification, version 2019, and followed up with an average of 24 months and these lesions have not changed.

### Patient demographics and mass features

In malignant cystic masses, the average BMI of patients was 23.9 ± 2.9, and in benign cystic masses, the average BMI of patients was 23.9 ± 3.5. The median size of malignant lesions was 4.2 cm (range: 1.4 cm - 12.2 cm), and the median size of benign lesions was 3.2 cm (range: 0.6 cm - 10.4 cm). The size of malignant lesions was significantly bigger than that of benign cystic masses (*P* = 0.005). There was no significant difference in gender, age, BMI of patients or location of tumor between benign and malignant lesions (*p* = 0.892, *p* = 0.181, *p* = 0.186, *p* = 0.777, respectively) (**Table 1**).

**Table 1:** Patient demographics and mass features between benign and malignant cystic renal masses.

	Benign	Malignant	*p* value
Gender^a^			0.892
Men	69 (73.4%)	26 (72.2%)	
Women	25 (26.6%)	10 (27.8%)	
Age (years)^b^	57 ± 13	53 ± 12	0.181
BMI (kg/m^2^)^a^			0.186
< 30	88 (93.6%)	36 (100%)	
≥ 30	6 (6.4%)	0 (0%)	
Tumor size (cm)^a^			0.005
< 5	57 (60.6%)	31 (86.1%)	
≥ 5	37 (39.4%)	5 (13.9%)	
Location^a^			0.777
Left	47 (50.0%)	19 (52.8%)	
Right	47 (50.0%)	17 (47.2%)	

BMI, body mass index.

a Chi-squared test, data are number (percentage).

b Student’s t test, data are mean ± SD.

### Characteristics of lesions on CT images

Of all lesions, 119 lesions had non-enhanced CT images and 114 lesions had enhanced CT images. CRMs characteristics on CT images are showed in **Table 2**. The incidences of cystic wall thickened, measurable enhancement of wall, more septa, measurable enhancement of septum, and wall nodule were significantly higher in malignant cystic masses than in benign masses (*p* = 0.005, *p* < 0.001, *p* < 0.001, *p* < 0.001, *p* < 0.001, respectively). However, there was no significant difference in calcification of any type or intracapsular density between the benign and malignant lesions (*p* = 0.721 and *p* = 0.448).

**Table 2:** Characteristics of lesions on CT images between benign and malignant cystic renal masses.

	Benign	Malignant	*p* value
Wall			0.005
< 4 mm	82 (89.1%)	18 (66.7%)	
≥ 4 mm	10 (10.9%)	9 (33.3%)	
Enhancement of wall			< 0.001
< 4 mm	85 (96.6%)	16 (61.5%)	
≥ 4 mm	3 (3.4%)	10 (38.5%)	
Septum			< 0.001
< 4	90 (97.8%)	18 (66.7%)	
≥ 4	2 (2.2%)	9 (33.3%)	
Enhancement of septum			< 0.001
< 4 mm	81 (92.0%)	12 (46.2%)	
≥ 4 mm	7 (8.0%)	14 (53.8%)	
Nodule(s)			< 0.001
None	89 (96.7%)	13 (48.1%)	
One or more	3 (3.3%)	14 (51.9%)	
Calcification			0.721
No	58 (63.0%)	16 (59.3%)	
Yes	34 (37.0%)	11 (40.7%)	
Intracapsular density			0.448
Homogeneous	85 (92.4%)	23 (85.2%)	
Heterogeneous	7 (7.6%)	4 (14.8%)	

Chi-squared test, data are number (percentage).

### Characteristics of lesions on MRI including DWI images

Of all lesions, 98 lesions had routine MRI images, 87 lesions had DWI images, and 50 lesions had enhanced MRI images. CRMs characteristics on MRI including DWI are presented in **Table 3**. The incidences of cystic wall thickened, measurable enhancement of wall, more septa, measurable enhancement of septum, wall nodule, high signal intensity of solid component on DWI, and heterogeneous intracapsular signal intensity were significantly higher in malignant cystic masses than in benign masses (*p* < 0.001, *p* < 0.001, *p* < 0.001, *p* < 0.001, *p* < 0.001, *p* < 0.001, *p* = 0.021, respectively).

**Table 3:** Characteristics of lesions on MRI including DWI images between benign and malignant cystic renal masses.

	Benign	Malignant	*p* value
Wall			< 0.001
< 4 mm	62 (88.6%)	13 (46.4%)	
≥ 4 mm	8 (11.4%)	15 (53.6%)	
Enhancement of wall			< 0.001
< 4 mm	28 (100%)	11 (50.0%)	
≥ 4 mm	0 (0%)	11 (50.0%)	
Septum			< 0.001
< 4	63 (90.0%)	8 (28.6%)	
≥ 4	7 (10.0%)	20 (71.4%)	
Enhancement of septum			< 0.001
< 4 mm	24 (85.7%)	7 (31.8%)	
≥ 4 mm	4 (14.3%)	15 (68.2%)	
Nodule(s)			< 0.001
None	69 (98.6%)	10 (35.7%)	
One or more	1 (1.4%)	18 (64.3%)	
Signal intensity on DWI			< 0.001
Low	63 (100%)	11 (45.8%)	
High	0 (0%)	13 (54.2%)	
Intracapsular signal intensity			0.021
Homogeneous	56 (80.0%)	16 (57.1%)	
Heterogeneous	14 (20.0%)	12 (42.9%)	

Chi-squared test, data are number (percentage).

**Figure 2:**
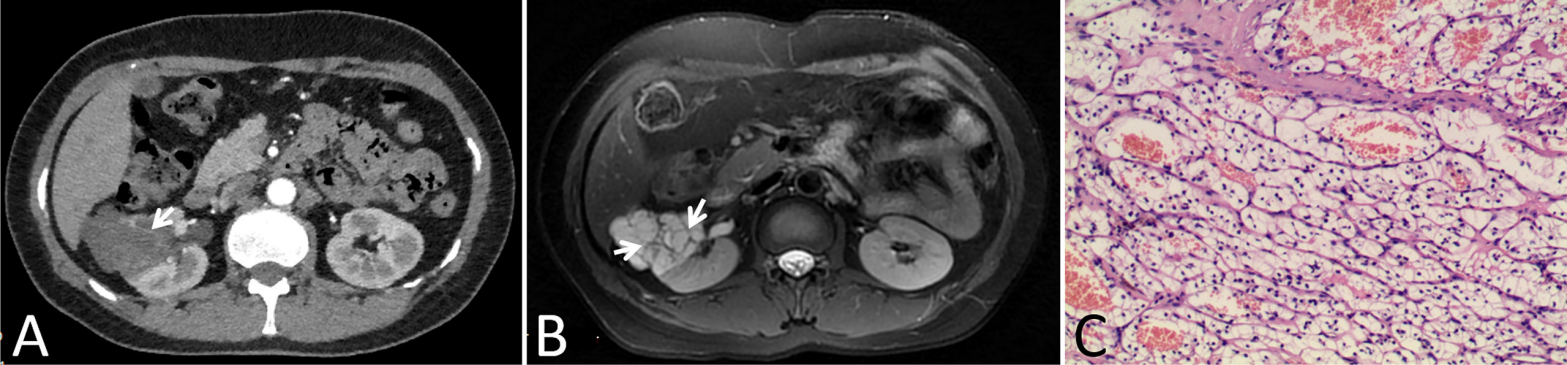
Images in a 46-year-old woman with a cystic mass in the right kidney. **(A)** Axial contrast-enhanced CT image shown a complex cystic mass that contained a few thin septa (arrow), **(B)** Axial T2-weighted MR image shown more septa (arrows) within the lesion than were depicted on the CT image, **(C)** This lesion was surgically removed and determined to be a renal clear cell carcinoma with cystic changes and glassy changes.

**Figure 3:**
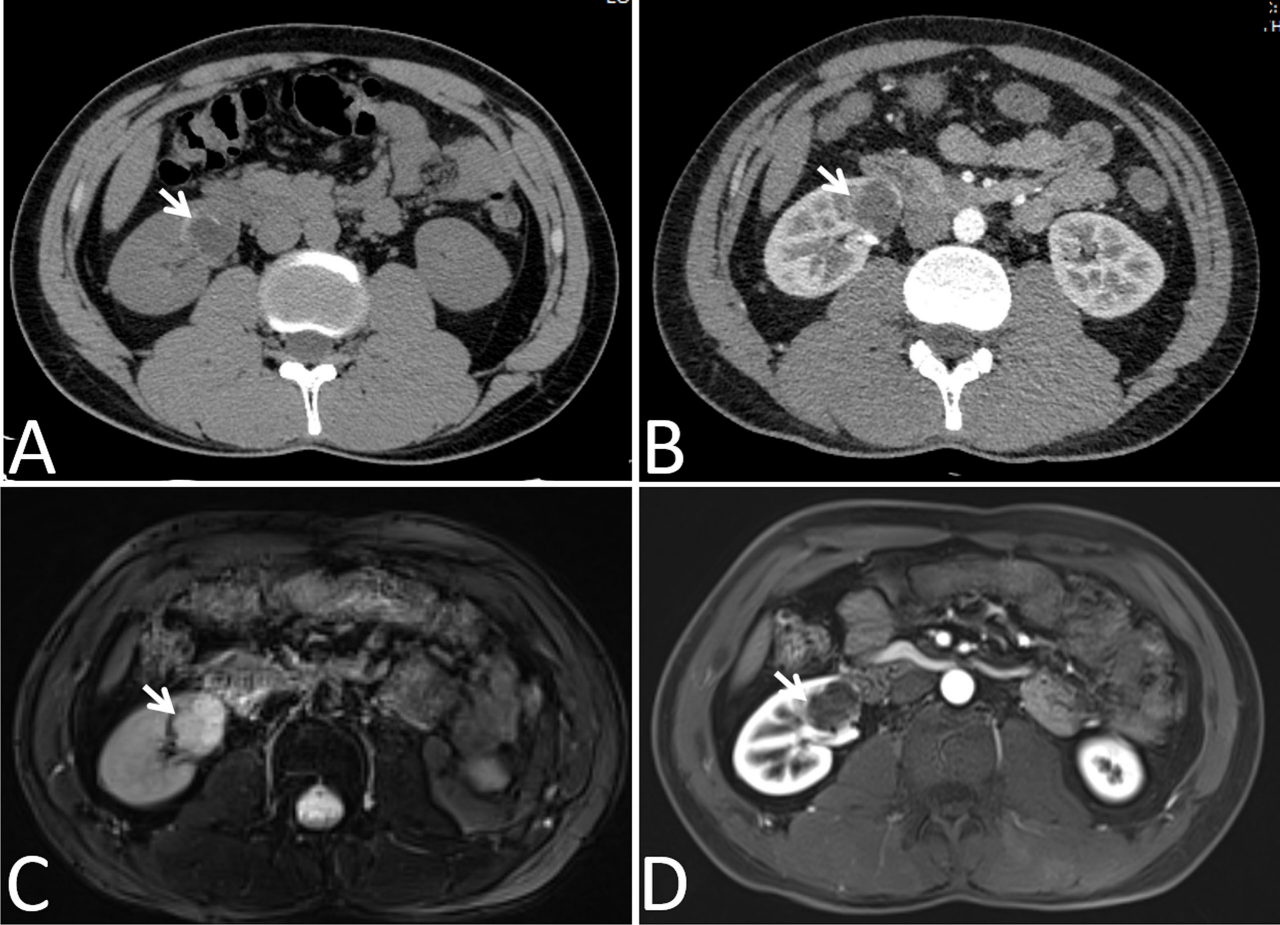
Images in a 24-year-old man had a cyst with calcium deposits in the right kidney confirmed by pathology. **(A)** Axial non-contrast-enhanced CT image shown a cystic mass with thick crescent calcification (arrow) in its wall, **(B)** Axial contrast-enhanced CT image shown the wall of cyst was obscured by the high density calcification (arrow), **(C)** Axial T2-weighted MR image shown irregular thickening of the lesion wall (arrows), **(D)** Axial gadolinium-enhanced fat-suppressed T1-weighted MR image shown no measurable enhancement of the wall (arrows). Calcification depicted on the CT image was not mistaken for enhancement on the MR image.

**Figure 4:**
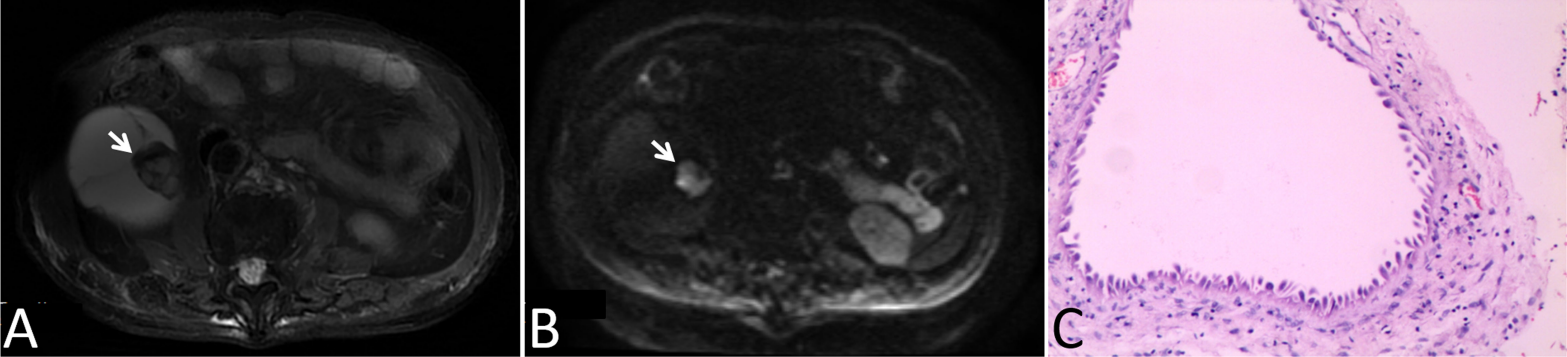
Images in a 78-year-old man with a cystic mass in the right kidney. **(A)** Axial T2-weighted MR image shown mural nodular soft tissue (arrow), **(B)** Axial DWI (b = 1000 s/mm2) image shown remarkably high signal intensity of the wall nodule (arrow), **(C)** This lesion was surgically removed and determined to be a multilocular cystic renal neoplasm of low malignant potential.

In our cohort, 87 lesions had both CT and routine MRI images, and MRI demonstrated more septa than did CT in 12 of 87 (13.8%) lesions (**Figure 2**). In one lesion, the cystic mass was classified as measurable enhancement of the wall on the enhanced CT because of the wall obscured by the high density calcification. Since calcification would not be depicted on the MRI, this suspected case of “pseudoenhancement” on CT has no measurable enhancement of the wall on enhanced MRI (**Figure 3**). Solid components of 13 cystic masses showed high signal intensity on DWI, all of which were malignant lesions (**Figure 4**).

### Diagnostic performance

**Figure 5:**
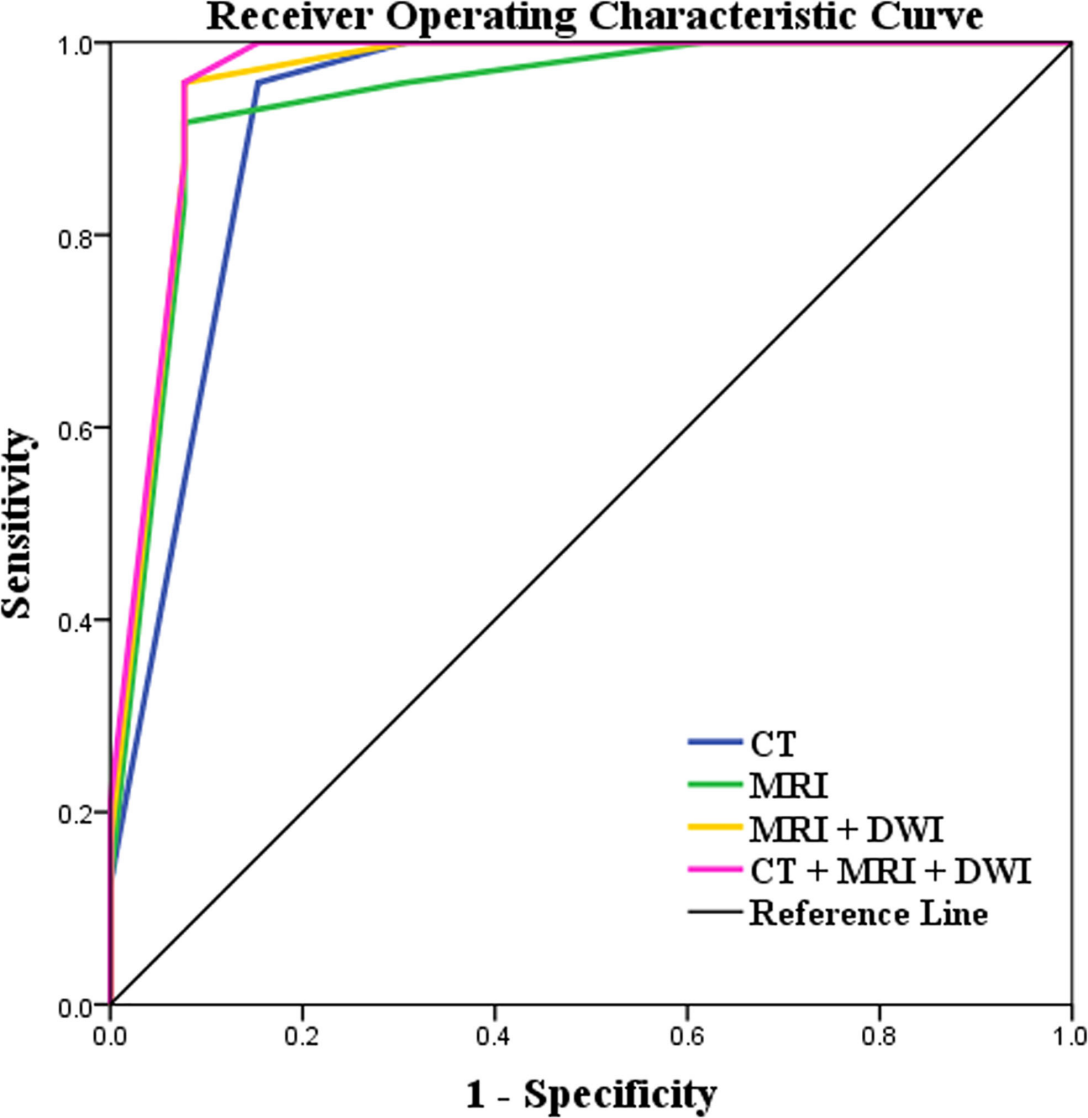
Receiver operating characteristic (ROC) curves of the characteristics of lesions on CT and MRI including DWI images for differentiating benign cystic renal masses from malignant. Combining MRI features including DWI and CT findings showed the highest AUC (AUC, 0.973; Sensitivity, 100%; Specificity, 92.3%) in distinguishing benign and malignant CRMs.

The diagnostic performances of CT and MRI including DWI on CRMs were detailed in **Table 4** and **Figure 5**. By ROC analysis, combining the above 7 significant image features on MRI including DWI generated a higher AUC (0.971) than combining the above 6 significant image features on MRI without DWI (AUC, 0.946) and combining the above 5 significant image features on CT (AUC, 0.926) for differentiating benign from malignant cystic masses. Combining MRI features including DWI and CT findings showed the highest AUC (0.973) for differentiating benign from malignant CRMs.

**Table 4:** Diagnostic performance of characteristics of lesions on CT and MRI including DWI images in differentiating benign cystic renal masses from malignant.

Parameters	AUC	Sensitivity (%)	Specificity (%)	Youden index	95% CI
CT	0.926	95.8	84.6	0.804	0.819-1.000
MRI	0.946	95.8	92.3	0.881	0.863-1.000
MRI+DWI	0.971	100	92.3	0.923	0.909-1.000
CT+MRI+DWI	0.973	100	92.3	0.923	0.914-1.000

AUC, area under the receiver operating characteristic curve; CI, confidence interval.

## Discussion

In our study, we found encouraging result that solid component of CRMs was high signal intensity on DWI and was likely to be characteristic of malignant tumors. The combination of the above significant MRI including DWI features with CT findings showed the highest AUC in distinguishing benign and malignant CRMs. These were similar to the results of previous studies (3, 5, 8, 9, 20). Pitra et al. reported that simple cysts showed homogenous signal with free diffusion, while complex cysts showed heterogeneous structure and restricted diffusion (5). DWI is a MRI technique that can be used to noninvasively quantify the diffusion of water molecules in biological tissues and provide additional histological features (21, 22). At present, DWI has been widely used in oncology research including renal cell carcinoma. The studies showed that DWI can help distinguish benign from malignant tumors (11, 15, 16). The cancer imaging RADS that used for diagnosis, monitoring, and treatment response evaluation have included DWI in the guidelines, such as PI-RADS, VI-RADS, and BI-RADS. Therefore, we suggest that the characteristics of CRMs on DWI should be incorporated into the Bosniak Classification.

It should be noted that some lesions were affected by intracapsular fluid and motion artifacts on DWI, leading to false positive or false negative results in the solid components of cystic masses (3, 4, 23, 24). In addition, apparent diffusion coefficient (ADC) is useful in tumor quantitative assessment and helps to remove T2 shine through effect in DWI (25–27). Inci et al. reported that ADC values of Bosniak III cysts were significantly lower than those of Bosniak I cysts (3). It may be due to the increase of solid components that restricting the motion of water molecules in tissue. At present, the first step of our research is qualitative research to explore the inclusion of DWI in the Bosniak classification criteria, and to directly obtain useful information to help distinguish benign and malignant lesions by review DWI images. In the future, we will further conduct post-processing analysis of DWI images and quantitative study based on ADC to explore whether quantitative DWI can help optimize the Bosniak classification.

In this study, CRMs showed thickened walls with measurable enhancement on CT images, which are more likely to be malignant tumors. Findings of some previous studies and the Bosniak classification (version 2019) have supported this (1, 8–10, 20, 28). At the same time, MRI also obtained similar results in our study. MRI has excellent soft tissue resolution, so it can better detect thickened walls (4, 5, 9, 29). In addition, sometimes it is difficult to determine whether the enhancement is present in lesion with calcification on CT (9, 29). In this case, MRI could serve to characterize these lesions, because calcification on MRI would not be misidentified or masked for enhancement. Therefore, wall thickened with enhancement has higher specificity on MRI than on CT (30).

Malignant cystic masses often appeared as several septa with enhancement on CT. This was also true on MRI and consistent with other studies and the Bosniak classification (version 2019) (1, 8–10, 20, 28). It may be due to better presented more internal septa with measurable enhancement on MRI. Therefore, several septa with enhancement were more sensitive on MRI than on CT. However, it should be noted that sometimes MRI artifacts owing to fluid fluctuation in capsule are mistaken for thickened septa or solid components (4).

In a recent study, wall nodules were significantly associated with malignant tumors, and complex cysts with wall nodules were more likely to progress over time (6). Similar to their findings and the Bosniak classification (version 2019), we found that wall nodules were more likely to occur in malignant cyst masses than in benign ones. In some cases, lesions showed enhanced wall nodules on MRI, while that only showed irregular septa on CT (4). However, because calcification appear as low signal intensity on MRI, nodular calcification is sometimes mistaken for a wall nodule. Therefore, it is necessary to combine image features of CRMs on CT for comprehensive evaluation (4, 5).

Unlike the study of Li et al, our results indicate that benign and malignant cystic masses have significant difference in intracapsular signal intensity on MRI, but there was no significant difference in intracapsular density on CT (1). These are similar to those mentioned in the Bosniak Classification, version 2019. Homogeneous CRMs on CT or MRI were classified as Bosniak I or Bosniak II masses, cystic masses that were heterogeneously hyperintense at unenhanced fat-saturated T1-weighted imaging on MRI were classified as Bosniak IIF masses (10). Benign lesions such as tuberculous granuloma and inflammatory focus would be misdiagnosed as malignant masses due to intracapsular density/signal intensity inhomogeneity. Therefore, the characteristic of cyst content may not predict malignancy accurately.

In our study, there was no significant difference in gender, age, or BMI between patients with benign and malignant CRMs. This was not completely consistent with previous studies (1, 2). The results of Goenka et al. showed that BMI was a predictor of Bosniak III cyst as a risk factor for malignancy (2). Obesity (BMI ≥ 30 kg/m^2^) is associated with epigenetic changes, and inflammation caused by obesity is associated with an increase in the amount of genotoxic reactive oxygen species. However, Asians are generally moderate in body type, with fewer obese people than Europeans and Americans. BMI may not be used as a risk factor to evaluate CRMs in Asians. As Silverman said, the proposed update to the Bosniak classification remains a malignancy prediction system, not a comprehensive management algorithm (10). Therefore, patient factors such as age, BMI, comorbidities, and risk tolerance all need to be considered in the personalized diagnosis and treatment plan, and recommendations for each Bosniak class.

Unexpectedly, the results of Goenka et al. showed that smaller Bosniak III cyst had a higher risk of being malignant (2). Although our study showed the size of malignant lesions was significantly bigger than that of benign cystic masses, the proportion of malignant masses with an average size of less than 5 cm is larger than that of benign masses. This was consistent with previous studies (1, 6, 31). Due to the popularity of imaging examinations, regardless of whether lesion is benign or malignant, the lesion is usually found earlier when it is small and may not have obvious characteristics. We agreed with Silverman et al. that mass size is not included in the Bosniak classification (version 2019). Mass size is related to behavior. Once a cystic mass has entered surveillance, the most important thing is not the overall change in size, but the change in morphology or the growth of solid elements over time (10, 32). Whether to include mass size or growth rate in the classification remains to be further studied.

There are some limitations in our study. Firstly, there were fewer patients who have both CT and MRI including DWI examinations in this single institution, retrospective study. To avoid unreliable results, we did not construct a model to predict the malignancy risk of CRMs. In the future, we will expand sample size to start this research. Secondly, the interval time between CT and MRI examinations of patients varied, and the secondary alterations of some cases between two scans may influence assessment. Thirdly, DWI obtained by SS-EPI used with a higher b-value had a lower SNR, resulting in image distortion. Our previous study on renal solid tumors found that DWI image quality of reduced field-of-view (r-FOV) was better, r-FOV DWI will be used to evaluate CRMs in the future (15). Finally, radiologist experience and interobserver variation were largely unexplored (30, 33).

## Conclusion

Our study found that DWI could help distinguish benign from malignant CRMs. The functional imaging sequence DWI can more precise imaging of inner microstructure and content of the lesions. Therefore, incorporating the characteristic of CRMs on DWI into the criteria can further optimize the Bosniak Classification, version 2019 to improve the assessment of CRMs.

## Data availability statement

The original contributions presented in the study are included in the article/supplementary material. Further inquiries can be directed to the corresponding author.

## Ethics statement

The studies involving human participants were reviewed and approved by Ethics Committee of Tongji Hospital, Tongji Medical College, Huazhong University of Science and Technology. Written informed consent for participation was not required for this study in accordance with the national legislation and the institutional requirements.

## Author contributions

AL designed the study, collected and analyzed the data, and wrote the manuscript. SL collected the data. YH analyzed the data. YS assisted with CT images evaluation and reviewed the final manuscript. XH assisted with MRI images evaluation and reviewed the final manuscript. DH supervised the work and reviewed the final manuscript. IK edited and reviewed the final manuscript. ZL supervised the work and made substantial contributions to the design of the study, reviewed the final manuscript. All authors contributed to the article and approved the submitted version.

## Funding

This study was supported by the National Natural Science Foundation of China (Grant No. 82071889, and No. 82071890).

## Conflict of interest

The authors declare that the research was conducted in the absence of any commercial or financial relationships that could be construed as a potential conflict of interest.

## Publisher’s note

All claims expressed in this article are solely those of the authors and do not necessarily represent those of their affiliated organizations, or those of the publisher, the editors and the reviewers. Any product that may be evaluated in this article, or claim that may be made by its manufacturer, is not guaranteed or endorsed by the publisher.
